# Pyroptosis in premature ovarian insufficiency: pathogenic mechanisms, pathway crosstalk, and therapeutic strategies

**DOI:** 10.3389/fmed.2026.1898429

**Published:** 2026-09-22

**Authors:** Yini Du, Jiamin Fu, Xiaoli Lai, Hanxiao Zhu, Kai Wang

**Affiliations:** 1 Department of Obstetrics and Gynecology, Taizhou Hospital of Zhejiang Province Affiliated to Wenzhou Medical University, Taizhou, Zhejiang, China; 2 State Key Laboratory for Diagnosis and Treatment of Infectious Diseases, and National Clinical Research Center for Infectious Diseases, The First Affiliated Hospital, School of Medicine, Zhejiang University, Hangzhou, China

**Keywords:** granulosa cells, NLRP3 inflammasome, pathway crosstalk, premature ovarian failure, pyroptosis, targeted therapy

## Abstract

Premature ovarian insufficiency (POI) is characterised by pathological loss of ovarian function in women younger than 40 years, resulting in premature exhaustion of the ovarian follicle pool and impaired fertility. Although apoptosis has long been considered the dominant mechanism driving granulosa cell (GC) death and follicular atresia, it does not fully explain the severe inflammatory microenvironment and tissue fibrosis observed during POI progression. Recently, pyroptosis, a proinflammatory form of programmed cell death mediated by the gasdermin family, has emerged as a critical driver of ovarian injury. This review comprehensively summarises the core mechanisms of pyroptosis across different POI aetiologies, with particular emphasis on the role of the NLRP3/caspase-1/GSDMD axis, the caspase-3/GSDME-mediated apoptosis-to-pyroptosis switch, and their multi-layered regulation by post-translational modifications, including S-palmitoylation, ubiquitination, and phosphorylation. We further discuss the complex molecular crosstalk between pyroptosis and other programmed cell death pathways, including apoptosis, ferroptosis, and autophagy, highlighting their synergistic roles in amplifying intraovarian inflammation. Finally, we evaluate emerging therapeutic strategies targeting pyroptosis, including mitochondrial reactive oxygen species scavengers, specific NLRP3/caspase-1 inhibitors, modulators of GSDMD post-translational modifications, and mesenchymal stem cell–derived exosome therapies. Collectively, these findings provide novel insights into POI pathogenesis and support the development of precise, non-hormonal therapeutic interventions to rescue the ovarian reserve and protect female reproductive health.

## 1 Introduction

### 1.1 Premature ovarian insufficiency (POI)

POI is a common clinical reproductive endocrine disorder defined as the loss of ovarian function in women before the age of 40 years (1). According to current guidelines, including those of the European Society of Human Reproduction and Embryology (2) and the National Institute for Health and Care Excellence (3), the diagnostic criteria for POI include menstrual irregularities, such as primary amenorrhoea or oligomenorrhoea, together with elevated levels of serum follicle-stimulating hormone. Notably, the serum follicle-stimulating hormone threshold used to define POI has undergone continuous refinement, having initially been set to 30 IU/L, subsequently lowered to 25 IU/L, and potentially subject to further reduction to 20 IU/L in the future (2).

Epidemiological surveys indicate that the prevalence of POI ranges from 1%–4% across various populations and is significantly influenced by regional, socioeconomic, and educational factors (4, 5). The premature decline of ovarian function not only results in the loss of fertility and subsequent infertility but also triggers a cascade of perimenopausal symptoms owing to the sustained hypoestrogenic state. This condition markedly increases the risk of long-term health complications, including osteoporosis, cardiovascular disease, neurodegenerative disorders, and psychological challenges such as anxiety and depression, thereby severely compromising the long-term well-being and quality of life of affected women (6, 7). Clinically, patients with POI frequently present with amenorrhoea, hot flashes, and vaginal dryness, although some may seek medical attention primarily because of infertility. Importantly, POI is not inherently irreversible; approximately 5%–10% of patients experience intermittent ovulation or even achieve natural conception, highlighting the dynamic and heterogeneous nature of its underlying pathological mechanisms (8).

The aetiology of POI is highly heterogeneous and involves a spectrum of factors, including genetic defects (9, 10), autoimmune diseases, iatrogenic factors (e.g., radiation and chemotherapy-induced damage) (11), environmental toxin exposure, and infections (12). However, more than half of these cases are idiopathic (13). Although the precipitating factors are complex, the core pathological mechanism underlying the development of POI consistently converges on exhaustion of the ovarian reserve. This specifically manifests as a reduction in the primordial follicle pool, accelerated follicular atresia, or impaired follicular growth, such as premature luteinisation, in which follicular cells undergo terminal differentiation into progesterone-producing luteal cells (14).

During follicular development, granulosa cells (GCs) facilitate material exchange and signal transduction with oocytes through gap junctions, thereby establishing the indispensable microenvironment required for oocyte growth and maturation. Current evidence indicates that the survival status of GCs is a critical determinant of follicular fate. Consequently, GC dysfunction and excessive cell death represent the key initiating steps in follicular atresia, which ultimately progresses to POI (15). Therefore, a thorough investigation of the specific molecular mechanisms governing GC death is essential for elucidating the fundamental nature of POI pathogenesis.

### 1.2 Limitations of current treatments

Currently, hormone replacement therapy (HRT) remains the cornerstone of POI management, aimed at alleviating hypoestrogenism-related symptoms, preventing osteoporosis, and improving the quality of life. However, HRT can neither restore ovarian reproductive function nor reverse the fundamental pathological process of follicular depletion. Furthermore, long-term HRT use is associated with potential risks, including thrombosis, breast cancer, and endometrial hyperplasia (8). In recent years, emerging strategies such as stem cell therapy, mitochondrial activators [e.g., *β*-nicotinamide mononucleotide (NMN)], and antioxidants (e.g., Coenzyme Q10) have demonstrated potential in animal models (15–17). Nevertheless, their clinical translation continues to face challenges, including variable efficacy, elusive mechanisms, and safety concerns. For instance, although human umbilical cord mesenchymal stem cells (hUCMSCs) have been shown to improve chemotherapy-induced POI by inhibiting ferroptosis (18), it remains unclear whether their effects involve other programmed cell death pathways. Therefore, an in-depth elucidation of the molecular mechanisms underlying follicular atresia and GC injury in POI, together with precise identification of key cell death patterns, has become an urgent prerequisite for the development of targeted intervention strategies.

### 1.3 Role of pyroptosis in POI

Previous research into the mechanisms of GC death has primarily focused on apoptosis, an immunologically 'silent' and non-inflammatory form of programmed cell death. Although apoptosis plays an essential role in follicular atresia, it does not fully account for the extensive inflammatory infiltration and fibrotic alterations observed in the ovarian microenvironment during the progression of POI (19). In recent years, pyroptosis—a pro-inflammatory form of programmed cell death—has emerged as a major research focus in reproductive medicine. Unlike apoptosis, pyroptosis is characterised by the assembly and activation of inflammasome complexes—such as nucleotide-binding oligomerisation domain-like receptor (NLR) family pyrin domain containing 3 (NLRP3) and absent in melanoma 2—which in turn mediate the activation of inflammatory caspases, including caspase-1. These activated caspases cleave gasdermin (GSDM) family proteins, such as Gasdermin D [GSDMD] and GSDME, to release the active N-terminal fragment (GSDM-NT). GSDM-NT subsequently translocates to the cell membrane and oligomerises to form pores of approximately 20 nm in diameter. The formation of these pores leads to osmotic cellular swelling and membrane rupture, thereby facilitating the robust release of pro-inflammatory cytokines, specifically interleukin-1β (IL-1β) and IL-18, and triggering a cascading inflammatory response (20).

In the field of reproductive medicine, aberrant activation of the NLRP3 inflammasome has been implicated in the pathological progression of polycystic ovary syndrome (PCOS) and ovarian cancer (21, 22). Recent studies have demonstrated that distinct pyroptotic features are detectable in ovarian GCs in models of POI induced by chemotherapy, environmental toxins, or autoimmune factors. This pyroptotic cell death not only directly leads to GC depletion but also releases inflammatory mediators that further disrupt the follicular microenvironment, thereby exacerbating injury to neighbouring cells. This process establishes a vicious cycle, positioning pyroptosis as a critical contributor to the onset and progression of POI (17).

Given the emerging significance of pyroptosis in POI pathogenesis, this review systematically summarises the current state of research in this field. We focus on the activation mechanisms of the NLRP3 inflammasome and its downstream effectors, including caspase-1 and GSDMD, in ovarian GCs and oocytes and explore how upstream signals—such as oxidative stress, DNA damage, and metabolic dysregulation—trigger this inflammatory death programme. Concurrently, this review integrates existing experimental evidence to evaluate the therapeutic potential of targeting pyroptotic pathways, including the use of NLRP3 inhibitors, exosome therapy, or NMN supplementation, to restore ovarian function. By elucidating the crosstalk between pyroptosis and other programmed cell death modalities, such as apoptosis and ferroptosis, this review aims to provide a theoretical foundation and identify novel molecular targets for the development of more precise and effective POI treatment strategies in the future (23, 24).

## 2 Core molecular mechanisms of pyroptosis

### 2.1 Overview of pyroptosis

Pyroptosis is a form of programmed cell death mediated by members of the GSDM family and is characterised by the robust release of pro-inflammatory mediators. The understanding of pyroptosis has evolved from initial phenotypic observations to the elucidation of its underlying molecular mechanisms. In 1992, Zychlinsky et al. first observed a rapid form of cell death in macrophages infected with *Shigella flexneri* that was distinct from classical apoptosis (25). In 2001, Cookson and Brennan coined the term “pyroptosis” to describe this caspase-1-dependent inflammatory mode of cell death (26). The identification of GSDMD as the key executioner of pyroptosis by Shao and Dixit in 2015 further established the molecular basis of this process and accelerated mechanistic studies (27, 28). In the human genome, the GSDM family comprises six principal members: GSDMA, GSDMB, GSDMC, GSDMD, GSDME (also known as DFNA5), and PJVK (also known as DFNB59). The murine GSDM family differs slightly from its human counterpart and includes GSDMA1–3, GSDMC1–4, GSDMD, GSDME, and PJVK but lacks GSDMB. Except for PJVK, these proteins possess a highly conserved structure consisting of two major functional domains: an N-terminal domain (also known as the pore-forming domain) and a C-terminal domain (also known as the repressor domain). The N-terminal domain is lipophilic and capable of binding membrane lipids, where it oligomerises to form pores in the plasma membrane. This domain is cytotoxic and functions as the principal effector moiety of pyroptosis. By contrast, the C-terminal domain primarily exerts an autoinhibitory function and is connected to the N-terminal domain through a flexible linker region. Under physiological conditions, intramolecular interactions between the N- and C-terminal domains maintain GSDM proteins in an inactive state (29).

Upon activation by upstream inflammatory signals, specific proteolytic cleavage within the linker region releases the N-terminal fragment (GSDM-NT), thereby exposing its membrane-binding ability. Once associated with the membrane, GSDM-NT undergoes oligomerisation to form nonselective hydrophilic pores with diameters of approximately 10–15 nm. These pores disrupt ionic homeostasis, causing water influx, cellular swelling, and eventual plasma membrane rupture. Consequently, intracellular components such as lactate dehydrogenase (LDH) are released, while mature inflammatory cytokines, particularly IL-1β and IL-18, are secreted into the extracellular environment. This combination of lytic cell death and inflammatory mediator release represents the defining feature distinguishing pyroptosis from other programmed cell death pathways (30).

### 2.2 Pyroptotic pathways

Pyroptosis execution relies on specific GSDM protein cleavage by inflammatory or apoptotic caspases. Driven by distinct upstream stimuli, pyroptosis occurs via canonical, non-canonical, caspase-3/GSDME, and alternative pathways.

#### 2.2.1 Canonical pathway

The canonical pathway is mediated by inflammasomes responding to pathogen-associated molecular patterns (PAMPs) or damage-associated molecular patterns (DAMPs). Upon activation, pattern recognition receptors (PRRs)—such as NLRP3, absent in melanoma 2 (AIM2), or Pyrin—recruit ASC (apoptosis-associated speck-like protein containing a caspase recruitment domain) to assemble the inflammasome complex and activate procaspase-1 (31–34). Active caspase-1 exerts dual functions: it cleaves GSDMD within its linker region (at Asp275 in humans) to liberate its pore-forming N-terminal domain (GSDMD-NT), while simultaneously processing pro-IL-1β and pro-IL-18 into mature cytokines (27, 35). GSDMD-NT insertion into the plasma membrane causes osmotic cell swelling, membrane rupture, and the extracellular release of mature IL-1β and IL-18, driving a robust inflammatory cascade (Figure 1A).

**Figure 1:**
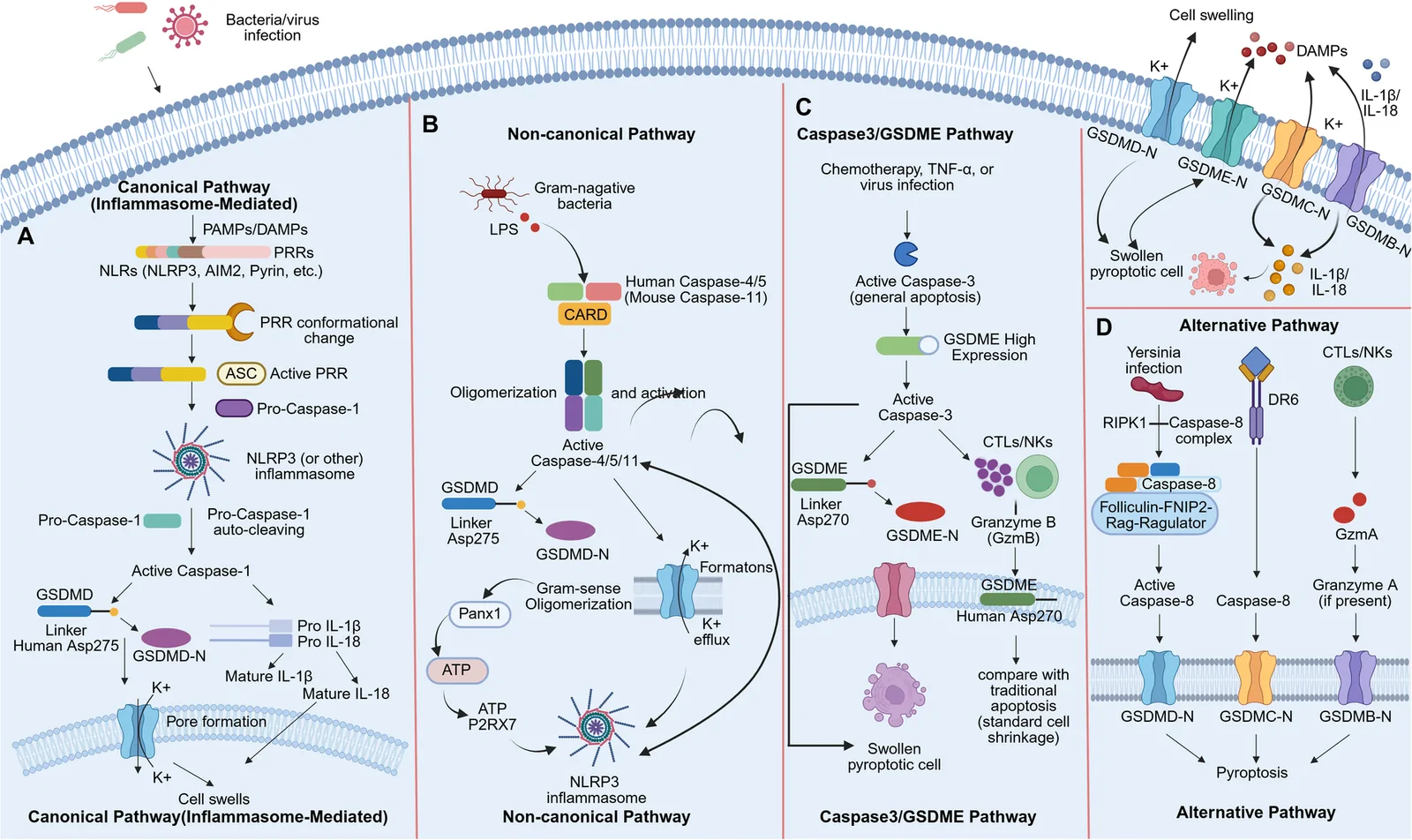
Overview of pyroptotic signaling pathways. **(A)** Canonical pathway: Intracellular PRRs sense PAMPs/DAMPs to assemble inflammasomes, activating caspase-1 to cleave GSDMD and process mature cytokines (IL-1β/IL-18). **(B)** Noncanonical Pathway: Cytosolic LPS directly activates human caspase-4/5 (murine caspase-11), triggering GSDMD-mediated pore formation and secondary NLRP3 activation via K+ efflux. **(C)** Caspase-3/GSDME pathway: Activated caspase-3 or Granzyme B cleaves GSDME in high-expression cells, effectively converting immunologically “silent” apoptosis into pro-inflammatory pyroptosis. **(D)** Alternative pathways: Atypical initiators, including caspase-8 and Granzyme A, mediate the cleavage of various Gasdermin members (GSDMD/C/B) to execute membrane rupture. Created in BioRender. Fu, J. (2026) https://BioRender.com/8zxor8x

#### 2.2.2 Noncanonical pathway

The Noncanonical Pathway serves as a primary defense against Gram-negative bacterial infections, initiated by direct sensing of intracellular lipopolysaccharide (LPS). Human caspase-4/5 and murine caspase-11 bind LPS directly via their CARD domains, triggering autocatalytic activation to cleave GSDMD and execute pyroptosis (27, 36, 37). Once activated, caspase-4/5/11 functions similarly to caspase-1 in that it directly cleaves GSDMD, generating the pore-forming GSDMD N-terminal fragment and thereby triggering pyroptotic cell death. However, unlike caspase-1, caspase-4/5/11 is incapable of directly processing the precursor cytokines pro-IL-1β and pro-IL-18 into their mature forms (38). In addition, caspase-4/5/11 cleaves the channel protein Pannexin-1, leading to ATP release and subsequent activation of the purinergic receptor P2X7. This signaling cascade further promotes activation of the NLRP3 inflammasome and induces pyroptosis (39). Furthermore, Potassium (K⁺) efflux resulting from GSDMD membrane pores secondarily engages the canonical NLRP3/caspase-1 axis, cooperatively promoting proinflammatory cytokine maturation and release (Figure 1B) (28, 38).

#### 2.2.3 Caspase-3/GSDME pathway

Traditionally, caspase-3 has been regarded as the principal executioner of apoptosis. However, recent studies have revealed that it also serves as a critical molecular switch in determining the mode of cell death and that its function is largely dependent on the intracellular expression level of GSDME. Under stimuli such as chemotherapeutic agents, tumour necrosis factor-α (TNF-α), or viral infection, activated caspase-3 specifically cleaves GSDME within its linker region in cells expressing high levels of GSDME, thereby generating the pore-forming GSDME N-terminal fragment and shifting the mode of cell death from apoptosis to pyroptosis (40).

Alternatively, cytotoxic T lymphocytes and natural killer cells secrete Granzyme B to directly cleave GSDME in target cells (Figure 1C). This mechanism not only provides a novel therapeutic target for cancer immunotherapy but also suggests that the extent of GSDME-mediated pyroptosis may serve as an important biomarker for predicting patient responsiveness to neoadjuvant immunotherapy (41).

#### 2.2.4 Alternative pathways of pyroptosis

Pyroptosis can also be engaged through alternative mechanisms. Under specific conditions, caspase-8 functions as an upstream initiator: during Yersinia infection, a metabolically regulated RIPK1 (receptor-interacting serine/threonine-protein kinase 1)/caspase-8 complex promotes GSDMD cleavage (42), while oxidized death receptor 6 signaling prompts caspase-8 to specifically cleave GSDMC, liberating its pore-forming N-terminal fragment (42). Alternatively, Granzyme A from cytotoxic T lymphocytes and natural killer (NK) cells directly cleaves GSDMB to release its cytotoxic N-terminal domain, executing pyroptotic cell death (Figure 1D) (43).

### 2.3 Post-Translational modifications (PTMs) of core components in pyroptosis

#### 2.3.1 S-Palmitoylation

S-palmitoylation serves as a critical mechanism for molecular targeting and membrane anchoring in pyroptosis. During initiation, ZDHHC family palmitoyl acyltransferases (particularly ZDHHC5 and ZDHHC7) modify NLRP3, promoting its trans-Golgi network recruitment and conformational interaction with NEK7 to facilitate inflammasome assembly (44–46).

During the downstream execution phase, microenvironmental stress signals, such as oxidative stress, stimulate ZDHHC5 and ZDHHC9 to covalently attach a 16-carbon palmitoyl group to a highly conserved cysteine residue in GSDMD (Cys191 in humans and Cys192 in mice) (47). This S-palmitoylation confers strong membrane affinity on the GSDMD N-terminal fragment, thereby enhancing its membrane insertion, oligomerisation, and pore-forming capacity (48, 49). Methodologically, due to the transient and dynamic nature of GSDMD lipid modification, highly sensitive techniques like acyl-biotin exchange and acyl-PEG exchange have become essential to overcome standard immunoblotting or mass spectrometry limitations, providing robust tools for tracking cysteine modification kinetics and evaluating palmitoylation-targeted therapeutics.

#### 2.3.2 Ubiquitination

Recent studies have demonstrated that GSDMD is regulated not only by inflammatory caspases but also by ubiquitination. Ubiquitination dynamically fine-tunes pyroptosis by either promoting activation or inducing degradation of core components. For instance, the E3 ligase SYVN1 catalyses the K27-linked polyubiquitination of GSDMD at Lys203 and Lys204, thereby enhancing GSDMD-mediated pyroptosis. Loss of SYVN1 markedly suppresses LDH release and plasma membrane rupture, indicating that SYVN1 functions as a positive regulator of GSDMD activation (50). In addition, TRIM21 has been reported to promote GSDMD ubiquitination, thereby enhancing its oligomerisation and pore-forming activity and amplifying inflammatory signaling (51).

In contrast to these activating forms of ubiquitination, GSDMD is subject to degradative ubiquitin-dependent regulation. Notably, the E3 ligase MIB2, in cooperation with the deubiquitinase USP18, mediates the ubiquitination of GSDMD at Lys168. Rather than promoting GSDMD activation, this modification serves as a signal for selective autophagic degradation. Overexpression of MIB2 significantly reduces GSDMD protein levels, whereas autophagy inhibitors such as chloroquine or bafilomycin A1 effectively block this degradation process (52). Furthermore, NEDD4L ubiquitinates both GSDMD and GSDME to regulate protein stability (53). Interestingly, pathogen-derived E3 ubiquitin ligases IpaH7.8 (secreted by *Shigella*) directly mediates polyubiquitination of human GSDMD, leading to its proteasomal degradation. This process suppresses host pyroptosis and facilitates bacterial infection (54).

In addition to regulating GSDM family proteins, ubiquitination plays a crucial role in controlling upstream inflammasome components. The E3 ubiquitin ligase TRIM31 catalyses K48-linked polyubiquitination of NLRP3, targeting it for proteasomal degradation to suppress inflammasome activation (55). Similarly, MARCH7 plays a key role in dopamine-induced signaling. Upon activation of the dopamine D2 receptor, MARCH7 mediates K48-linked ubiquitination of NLRP3, which not only reduces intracellular NLRP3 abundance but also limits excessive inflammasome assembly (56). In addition to these mechanisms, ARIH2 (interacting with the NACHT domain of NLRP3) and RNF125 function as negative regulators of NLRP3 (57, 58). Collectively, these E3 ligases establish a multilayered negative feedback network that prevents excessive NLRP3 accumulation and aberrant pyroptotic activation.

#### 2.3.3 Phosphorylation

Phosphorylation acts as a vital molecular switch regulating both executioners and upstream modulators of pyroptosis. AMP-activated protein kinase (AMPK) can directly phosphorylate the GSDMD N-terminal fragment at Ser46, thereby impairing its oligomerisation and pore-forming capacity. This modification ultimately suppresses pyroptosis and attenuates the antitumour effects (59).

Phosphorylation of NLRP3 is a crucial rate-limiting step in its activation. JNK1-mediated phosphorylation of NLRP3 at Ser194 (corresponding to Ser198 in mice) is essential for inflammasome assembly. This modification relieves the autoinhibitory conformation of NLRP3 and promotes its recruitment to the adaptor protein ASC (60). In addition, protein kinase D (PKD) phosphorylates NLRP3 at Ser293 on mitochondria-associated endoplasmic reticulum (ER) membranes or the Golgi apparatus, thereby driving the conformational transition of NLRP3 from an autoinhibited state to an active state. This enables NLRP3 to dissociate from membrane structures and translocate to the cytosol for inflammasome assembly (61). More importantly, PKD phosphorylates the critical NLRP3-interacting protein NEK7. Recent studies have shown that, following GSDMD pore formation and the resulting K⁺ efflux, intracellular PKD becomes activated and phosphorylates NEK7 at Ser204. This phosphorylation event stabilises the interaction between NEK7 and NLRP3, thereby further amplifying NLRP3 inflammasome assembly and activity downstream of K⁺ efflux and GSDMD activation. This NEK7 phosphorylation-dependent feedback mechanism provides a mechanistic explanation for the rapid escalation of localised membrane pore formation into a systemic inflammatory response (62). By contrast, NLRP3 phosphorylation at Ser295 by PKA exerts an inhibitory effect by suppressing ATPase activity, thereby dampening inflammasome activation and inflammatory responses (63). Collectively, targeting these phosphorylation checkpoints provides multi-tiered therapeutic strategies for controlling pyroptosis-associated inflammatory diseases.

## 3 Pathological links between pyroptosis and POI

### 3.1 Inflammation and oxidative stress in the ovarian microenvironment

The maintenance of ovarian microenvironmental homeostasis is essential for normal follicular development. During the onset and progression of POI, excessive accumulation of reactive oxygen species (ROS) and chronic low-grade inflammation within the ovarian microenvironment together create a pathological milieu conducive to the induction of pyroptosis (64). Under physiological conditions, moderate levels of ROS are required for normal follicular development and intracellular signaling (65). However, exogenous physicochemical insults or endogenous aging can lead to the excessive production of ROS in the ovaries. These free radicals not only directly induce lipid peroxidation and DNA damage but also function as critical upstream stress signals that trigger NLRP3 inflammasome activation (66). Rather than acting as classical, direct structural DAMPs, ROS facilitate NLRP3 assembly primarily through several indirect intermediaries and organelle-level damage (67). Specifically, excessive mitochondrial ROS (mtROS) induce mitochondrial outer membrane permeabilization, causing the leakage of oxidized mitochondrial DNA (ox-mtDNA)—a canonical cytosolic DAMP—into the cytoplasm where it directly engages NLRP3 (68, 69). Concurrently, mtROS elevate cellular oxidative stress to promote the dissociation of thioredoxin-interacting protein (TXNIP) from thioredoxin (TRX), enabling free TXNIP to bind NLRP3 and drive inflammasome assembly (70, 71). Additionally, ROS can induce oxidative post-translational modifications at specific cysteine residues on NLRP3 or its essential partner NEK7 (72, 73). Although whether ROS act predominantly via indirect organelle dysfunction/ox-mtDNA release or direct oxidative conformational shifts remains an area of active debate (74), mtROS-driven signaling cascades consistently represent an early, essential checkpoint for pyroptosis in ovarian resident macrophages and GCs (75). Upon activation, NLRP3 recruits ASC and caspase-1 to form the inflammasome complex, thereby directly initiating the canonical pyroptotic pathway and promoting the maturation and release of pro-inflammatory cytokines, such as IL-1β and IL-18 (76). Meanwhile, locally released primary inflammatory mediators, including TNF-α, further amplify this response. This self-perpetuating positive feedback loop—characterised by oxidative stress, inflammasome activation, and pyroptosis—disrupts ovarian immune tolerance and maintains the ovarian microenvironment in a state of chronic low-grade inflammation, thereby laying the foundation for subsequent widespread damage to ovarian parenchymal cells.

### 3.2 Pyroptosis of GCs

GCs are essential somatic cells that surround oocytes, provide metabolic support, and secrete oestrogen. The survival status of GCs is a decisive determinant of follicular fate. During the pathological progression of POI, particularly in chemotherapy-induced ovarian injury [e.g., cisplatin or cyclophosphamide (CTX) exposure], extensive pyroptotic death of GCs has been observed. Recent studies have demonstrated that chemotherapeutic agents, as well as excessive accumulation of ROS within the ovarian microenvironment, act as potent stress signals that lead to aberrant activation of the NLRP3 inflammasome in GCs. Activated NLRP3 subsequently recruits caspase-1 and cleaves GSDMD, resulting in plasma membrane pore formation and the execution of canonical pyroptosis. Given that GCs are tightly interconnected with oocytes and other somatic cells through gap junctions, pyroptotic death of GCs disrupts metabolic support and intercellular signaling to the oocyte, thereby depriving it of a supportive survival niche. In a mouse model of PCOS, significant activation of the caspase-1/GSDMD axis in GCs was reported, accompanied by increased follicular atresia. Notably, pharmacological inhibition of pyroptosis using agents such as disulfiram effectively reduced GC death and improved follicular development (77). When GCs undergo swelling, membrane rupture, and pyroptotic lysis, not only is the metabolic supply to the oocyte interrupted, but a pronounced “bystander effect” is also triggered, leading to the collapse of the entire follicular structure. This process directly accelerates follicular atresia and contributes to irreversible depletion of the ovarian reserve. Conversely, genetic ablation or pharmacological inhibition of the NLRP3 inflammasome and downstream pyroptotic pathways in GCs has been shown to effectively preserve the ovarian reserve and delay ovarian aging (76, 78).

### 3.3 Decline in oocyte quality

The quality of oocytes is highly dependent on the homeostasis of the surrounding microenvironment. Pyroptosis-induced inflammatory cascades can directly impair oocyte maturation and fertilisation potential. Pro-inflammatory cytokines released from pyroptotic GCs, including IL-1β and IL-18, can diffuse into the cumulus–oocyte complex and activate stress-related signaling pathways within the oocyte. In PCOS models, GC pyroptosis has been shown to induce significant upregulation of ER stress markers in oocytes, such as GRP78 and CHOP, accompanied by abnormal spindle morphology and disrupted chromosomal alignment. These alterations ultimately reduce oocyte maturation rates and impair embryonic developmental competence (77, 79). Exposure to high concentrations of inflammatory mediators further induces severe ER stress and mitochondrial dysfunction in oocytes (80). As mitochondria serve as the primary energy-producing organelles of oocytes, their impairment results in insufficient ATP production, which in turn causes meiotic abnormalities, defective spindle assembly, and an increased rate of chromosomal aneuploidy. Furthermore, prolonged exposure to an IL-1β–rich inflammatory microenvironment can disrupt epigenetic regulation in oocytes, markedly compromising fertilisation capacity and subsequent embryonic development potential (81). Collectively, pyroptosis-mediated disruption of the inflammatory ovarian microenvironment represents a central mechanism underlying poor oocyte quality and reduced fertility in patients with POI.

### 3.4 Microbiota-derived metabolites as emerging regulators of ovarian inflammatory homeostasis

Increasing evidence suggests that the gut and vaginal microbiota represent important regulators of reproductive health by shaping systemic and local inflammatory environments (82, 83). Microbial dysbiosis can influence host immunity through alterations in microbiota-derived metabolites, including short-chain fatty acids (SCFAs), bile acid derivatives, and tryptophan metabolites, thereby affecting inflammatory signaling and tissue homeostasis (63).

Among these metabolites, SCFAs, including acetate, propionate, and butyrate, have emerged as important immunomodulatory molecules (84). SCFAs regulate immune cell function through G-protein-coupled receptors, such as GPR43/FFAR2 and GPR109A, and through epigenetic regulation via histone deacetylase inhibition (85). These pathways contribute to macrophage homeostasis and suppress excessive inflammatory responses.

Microbiota-derived metabolites have also been implicated in the regulation of inflammasome activation (86). Alterations in microbial composition and metabolite availability can influence mitochondrial metabolism, macrophage polarization, and redox signaling, thereby modulating NLRP3 inflammasome activity (70, 74). For example, disruption of metabolic homeostasis and excessive oxidative stress can promote mitochondrial dysfunction and downstream NLRP3 activation (70).

In addition, microbial tryptophan metabolites regulate host immunity through aryl hydrocarbon receptor (AhR)-dependent pathways, which influence inflammatory cell differentiation and tissue immune balance (87). Although direct evidence linking microbiota dysbiosis to ovarian NLRP3-GSDMD-mediated pyroptosis remains limited, these findings suggest that the microbiota–metabolite–immune axis may represent an important upstream regulator of ovarian inflammatory injury and may contribute to pyroptosis-associated POI.

Given the emerging role of protein post-translational modifications in pyroptosis regulation (47), whether microbiota-derived metabolites can influence ZDHHC-mediated protein palmitoylation represents an intriguing but largely unexplored area requiring further investigation.

## 4 Specific mechanisms of pyroptosis in POI of different aetiologies

### 4.1 Iatrogenic/chemotherapy-induced POI

Chemotherapy is one of the most common causes of iatrogenic POI in young women. Exposure to chemotherapeutic agents, particularly alkylating agents such as CTX and platinum-based compounds such as cisplatin, elicits profound stress responses within the ovarian tissue. First, these agents induce massive production of ROS within the ovarian microenvironment. This severe oxidative stress serves as a potent source of DAMPs, which directly and excessively activate the NLRP3 inflammasome in GCs. Activated NLRP3 subsequently recruits caspase-1, leading to GSDMD cleavage and induction of canonical GSDMD-mediated pyroptosis, accompanied by the release of pro-inflammatory cytokines. This process ultimately results in extensive follicular atresia (17). In addition, at the nuclear level, severe DNA damage induced by chemotherapeutic agents strongly activates caspase-3, a protease traditionally associated with apoptotic signaling. Because normal ovarian tissues, particularly GCs, express high levels of GSDME, activated caspase-3 can redirect its substrate specificity from canonical apoptotic targets to GSDME. This results in the specific cleavage of GSDME and release of its pore-forming N-terminal fragment. Consequently, DNA damage drives a molecular switch from apoptosis to pyroptosis, transforming what would otherwise be a relatively immunologically silent apoptotic process into a highly destructive inflammatory form of cell death. The apoptosis-to-pyroptosis transition has been established as a central mechanism underlying chemotherapy-induced ovarian toxicity (40).

### 4.2 Autoimmune/inflammatory POI

Approximately 20% of POI cases are associated with autoimmune mechanisms and are frequently accompanied by other autoimmune disorders such as Addison's disease and Hashimoto's thyroiditis. During the initiation and progression of autoimmune POI, disruption of immune tolerance leads to abnormal infiltration of large numbers of inflammatory immune cells, including macrophages and lymphocytes, into the ovarian tissue. These aberrantly activated immune cells release substantial amounts of mature pro-inflammatory cytokines, such as IL-1β and IL-18, into the local ovarian microenvironment through excessive activation of the NLRP3/caspase-1 signaling pathway. These high concentrations of inflammatory mediators not only directly impair follicular development but also stimulate ovarian somatic cells, thereby triggering secondary pyroptosis. This process establishes a vicious local cycle characterised by immune cell infiltration, cytokine storms, and pyroptotic death of ovarian cells (88). Anti-ovarian autoantibodies, such as anti-GC antibodies, specifically recognise and attack ovarian GCs and oocytes. The formation of antigen–antibody complexes and subsequent activation of the complement system serve as potent surface stress signals, further synergistically inducing membrane pore formation and extensive pyroptosis in GCs. This accelerates ovarian parenchymal destruction and ultimately leads to progressive ovarian functional failure (89).

### 4.3 POI induced by genetic and environmental factors

Genetic defects and environmental exposure are critical triggers of POI. Mutations in mitochondrial DNA (mtDNA) or abnormalities in nuclear genes, such as *POLG* and *CPEB1*, can lead to mitochondrial respiratory chain dysfunction, impaired ATP synthesis, and massive accumulation of ROS (90, 91). Persistent oxidative stress damages mitochondria and induces ER stress, thereby activating the unfolded protein response (UPR). When the UPR fails to restore protein proteostasis, the PERK/eIF2*α*/ATF4 pathway undergoes sustained activation and upregulates CHOP expression, which subsequently promotes the assembly of the NLRP3 inflammasome (92). Multi-omics analyses have demonstrated that genes associated with oxidative phosphorylation, such as COX5A and UQCRFS1, are significantly downregulated in the ovarian tissues of patients with POI, whereas genes involved in inflammatory and apoptotic pathways are upregulated. These findings suggest that the mitochondrial–ER–pyroptosis axis plays a pivotal role in the pathogenesis of hereditary POI (93–95). Furthermore, widespread exposure to endocrine-disrupting chemicals and environmental toxins, such as microplastics and bisphenol A, poses a severe threat to female reproductive health (96). Recent environmental toxicological studies have confirmed that specific concentrations of pollutants, such as polystyrene microplastics, can enter the ovarian microenvironment and trigger intense oxidative stress in ovarian GCs. This, in turn, overactivates the NLRP3/caspase-1 signaling pathway, directly triggering GSDMD-mediated pyroptosis (97). This mechanism results in a precipitous decline in the number of growing follicles and premature exhaustion of the ovarian reserve (78).

### 4.4 Clinical evidence and human data

#### 4.4.1 Follicular fluid microenvironment

In the clinical practice of assisted reproduction, follicular fluid (FF) is regarded as the “gold standard” specimen for assessing the oocyte developmental microenvironment. Current clinical indicators for POI, including serum FSH, AMH, and antral follicle count, mainly reflect ovarian reserve but provide limited information regarding inflammatory injury and molecular mechanisms. Clinical cohort studies focusing on patients with diminished ovarian reserve and early-stage POI have demonstrated that the concentrations of IL-1β and IL-18 in both FF and peripheral blood are significantly higher than those observed in healthy age-matched women (66, 98). Crucially, follicular fluid represents a clinically accessible microenvironment containing inflammatory and stress-associated molecular signatures. Current evidence indicates that pyroptosis-related components (99, 100), inflammasome activation markers (66), and mitochondrial damage-associated molecules (98) may contribute to ovarian inflammatory pathology, although their value as liquid biopsy biomarkers in POI remains to be systematically investigated. Clinical metabolomics and molecular blotting analyses of FF from patients with reproductive disorders and chronic low-grade inflammation have further identified direct upstream signals that drive pyroptosis. Specifically, abnormally elevated levels of free fatty acids, such as palmitic acid, may serve as critical lipid donors within the follicular microenvironment. These free fatty acids drive and exacerbate the S-palmitoylation of GSDMD within GCs, thereby inducing irreversible cellular pyroptosis (99). These findings elucidate a crucial pathological mechanism through which metabolic dysregulation activates pyroptotic executioner proteins via specific PTMs. Importantly, future biomarker development in POI may benefit from moving beyond conventional measurements of protein abundance toward functional signatures determined by post-translational modifications (PTMs). Since pyroptotic execution is regulated not only by the expression of inflammasome components but also by dynamic modifications controlling protein activation, localization, and stability, PTM-associated signatures of key pyroptotic regulators, such as GSDMD palmitoylation, ubiquitination, or oxidative modifications, may provide mechanistic information complementary to traditional inflammatory biomarkers. Although such PTM-based biomarkers have not yet been clinically validated in POI, their development may offer a strategy to identify patients with active inflammatory ovarian injury and monitor therapeutic responses. Although circulating GSDMD-N has not yet been systematically evaluated in patients with POI, accumulating evidence demonstrates that GSDMD activation generates extracellular inflammatory signatures, including GSDMD-associated vesicular structures and released pyroptotic components. These findings raise the possibility that GSDMD-derived molecules may serve as future indicators of pyroptotic activity and disease stratification in POI (101–103).

#### 4.4.2 Association between molecular characteristics of human primary GCs and *in vitro* fertilisation (IVF) outcomes

Primary cell data derived directly from patients further substantiate the occurrence of pyroptosis in human ovarian ageing, which is characterised by an inflammation-ageing phenotype. Analyses of luteinised human primary GCs collected during IVF oocyte retrieval procedures and multiple transcriptomic and proteomic studies have revealed pathological upregulation of NLRP3, caspase-1, and the executioner protein GSDMD in POI patients compared with controls (104). This cell-type-specific hyper-activation directly correlates with oocyte oxidative damage, reduced maturation rates, and elevated early embryo fragmentation. Furthermore, extensive retrospective cohort studies from reproductive centres have demonstrated that this localised chronic activation of the NLRP3 inflammasome and subsequent pyroptosis within follicles exhibits a highly significant positive clinical correlation with oocyte oxidative damage, impaired maturation rates, and elevated early embryo fragmentation during the IVF cycle (100).

#### 4.4.3 Mendelian randomisation (MR) and causal inference in systems genetics

From the perspective of systems genetics, large-scale genome-wide association studies provide high-level evidence supporting a causal relationship between systemic inflammation, pyroptosis, and POI. A bidirectional MR study systematically evaluated 41 systemic inflammatory regulators and confirmed a significant causal association between the aberrant expression of specific inflammatory cytokines, such as IL-33, and the risk of developing POI at the genomic level (105). Another recent MR cohort study conducted in large European populations further demonstrated that genetically elevated levels of circulating IL-18, a key downstream effector of pyroptosis, together with its receptor IL-18R1, serves as independent core risk factors driving genetic susceptibility to POI in women (106). Collectively, these findings from extensive human epidemiological cohorts effectively transcend species-specific limitations and substantiate the significant potential of targeted interventions within the pyroptotic-inflammatory network for the clinical prevention and management of human POI.

#### 4.4.4 Circulating biomarkers and exosomal cargo

Beyond localized fluids, systemic circulating biomarkers offer immense early-warning potential (107, 108). Extracellular vesicles, particularly circulating serum exosomes, stably encapsulate molecular signatures from the damaged intraovarian stroma (109). Specific exosomal miRNAs and bioactive cargoes (such as miR-21-5p) that post-transcriptionally target the NLRP3 inflammasome axis and pyroptotic mediators exhibit significant dysregulation in POI contexts, directly reflecting intraovarian pyroptotic stress and providing promising non-invasive diagnostic indicators (110, 111).

Collectively, current human studies provide initial evidence that pyroptotic activation is not merely an experimental phenomenon but is associated with clinical manifestations of ovarian dysfunction. From a translational perspective, circulating pyroptosis-associated molecules may represent promising biomarkers for early identification and stratification of POI. Potential candidates include IL-1β, IL-18, pyroptosis-related components, and extracellular vesicle-associated inflammatory signatures. However, compared with cancer and autoimmune diseases, systematic evaluation of pyroptosis-derived biomarkers in POI remains largely unexplored, representing a major unmet clinical need.

### 4.5 Physiological considerations and therapeutic window of pyroptosis inhibition

Although excessive NLRP3 inflammasome activation contributes to ovarian inflammatory injury and follicular depletion, inflammasome signaling should not be considered exclusively pathogenic (17, 112). Physiological inflammatory responses are required for normal ovarian processes, including ovulation, follicular rupture, extracellular matrix remodeling, and immune cell recruitment (113). Therefore, complete or prolonged suppression of NLRP3 activity may potentially interfere with normal ovarian homeostasis (35, 114).

Consistent with this concept, therapeutic inhibition of pyroptosis should focus on restoring the balance between pathological and physiological inflammatory signaling rather than achieving complete inflammasome suppression (66, 115). The optimal therapeutic strategy may depend on disease stage, as transient inhibition during excessive inflammatory activation may preserve follicular reserve, whereas prolonged blockade in individuals with preserved ovarian function may theoretically disrupt normal reproductive processes (67, 76). Future studies should therefore carefully evaluate reproductive safety, treatment duration, and patient stratification before clinical application of pyroptosis-targeting therapies (116). Despite the diverse etiological classifications of POI, including genetic abnormalities, autoimmune disorders, iatrogenic injury, and environmental insults, increasing evidence suggests that these distinct pathological processes may converge on several common upstream stress signals (2). Among them, oxidative stress represents a central integrative mechanism linking different POI etiologies to ovarian dysfunction (64, 65).

Excessive accumulation of ROS can impair mitochondrial function, induce DNA damage, disrupt follicular cell metabolism, and alter ovarian immune homeostasis (75, 117). These stress responses may subsequently activate multiple regulated cell death pathways, including apoptosis, pyroptosis, ferroptosis, and autophagy-associated dysfunction (24, 118, 119). Therefore, rather than acting as independent events, different forms of ovarian cell death may represent interconnected downstream consequences of shared upstream stressors (120).

This convergence provides a mechanistic framework for understanding how diverse POI triggers ultimately result in follicular depletion and ovarian insufficiency, and highlights oxidative stress and mitochondrial dysfunction as potential therapeutic entry points for restoring ovarian homeostasis (100).

## 5 Molecular crosstalk between pyroptosis and other cell death modalities in POI

Within the intricate pathological network of POI, depletion of ovarian GCs is not an isolated event driven by a single pathway. Instead, a profound biochemical crosstalk exists between pyroptosis and other forms of regulated cell death, including apoptosis, ferroptosis, and autophagy. These death programmes share critical metabolic nodes and kinase switches that, under specific stress conditions, can undergo mutual transformation, synergistic interaction, or cascade amplification, ultimately leading to the comprehensive collapse of follicular microenvironmental homeostasis.

### 5.1 Pyroptosis and apoptosis

Apoptosis has long been regarded as the immunologically silent pathway for follicular atresia. However, during the pathological progression of iatrogenic POI, members of the caspase family function as critical molecular switches that determine the mode of cell death. In chemotherapy-induced ovarian injury, such as that caused by cisplatin or CTX, severe DNA damage during the early stages of treatment triggers an intracellular apoptotic cascade and extensively activates the classical executioner caspase-3. Given that ovarian GCs naturally express high levels of GSDME, a rapid increase in activated caspase-3 beyond a critical threshold shifts its substrate preference towards specific cleavage of the flexible linker region of GSDME, thereby releasing the pore-forming GSDME-N fragment (Figure 2A). This mechanism converts immunologically silent apoptosis into pro-inflammatory pyroptosis (40). Recent studies have highlighted that ovarian tissue damage is no longer considered to result solely from apoptosis; rather, it involves sophisticated crosstalk among multiple programmed death pathways, including pyroptosis and ferroptosis. This may explain why ovarian tissue exhibits massive cellular loss and irreversible diffuse inflammatory fibrotic injury following chemotherapy or exposure to other pathological stressors (118).

**Figure 2:**
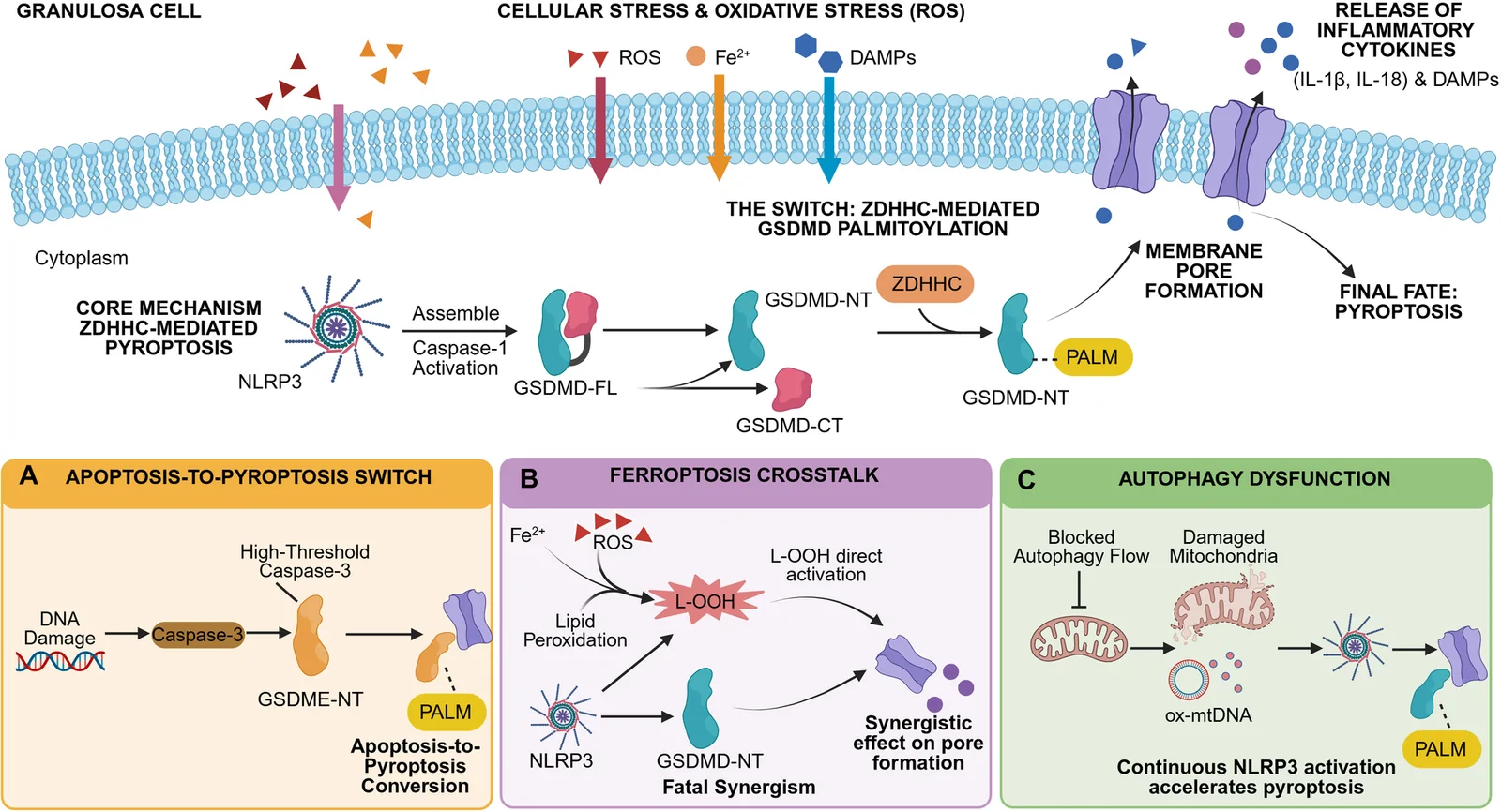
Molecular crosstalk between pyroptosis and other cell death modalities in granulosa cells. **(A)** Apoptosis-to-Pyroptosis Switch: Severe DNA damage triggers high-threshold Caspase-3 activation, which cleaves GSDME to redirect the cell death fate from immunologically silent apoptosis toward pro-inflammatory pyroptosis. **(B)** Ferroptosis Crosstalk: Fe2+-driven lipid peroxidation (L-OOH) provides direct activation signals that synergize with GSDMD to accelerate membrane rupture. **(C)** Autophagy Dysfunction: Blocked autophagic flux leads to the accumulation of damaged mitochondria and ox-mtDNA, which persistently activates the NLRP3 inflammasome and accelerates the pyroptotic program. Created in BioRender. Fu, J. (2026) https://BioRender.com/r4xn5j0

Beyond classical pyroptotic pathways, caspase-8 exhibits a highly flexible “molecular bypass” capability. Under specific conditions of pathological stress or death receptor activation, activated caspase-8 can completely bypass the traditional caspase-1 inflammasome axis to directly cleave GSDMD and GSDMC proteins. This non-canonical substrate cleavage pathway enables cells to rapidly initiate highly destructive atypical pyroptosis under extreme stress conditions, thereby expanding the conceptual boundaries of pyroptosis initiation mechanisms (121, 122).

More importantly, these distinct caspase-mediated death pathways do not exist in a simple “either–or” state during the fate determination of ovarian GCs; instead, they demonstrate a synergistic amplification effect. *In vitro* pharmacological intervention experiments provide robust biochemical evidence supporting this profound crosstalk. When the human ovarian GC line KGN is treated with cisplatin, strong apoptosis and pyroptosis signaling cascades are simultaneously activated within the cells. Experimental data demonstrated that, although the caspase-3 inhibitor Z-DEVD-FMK partially reduced LDH release in the culture supernatant by suppressing the apoptotic pathway, it failed to completely prevent extensive plasma membrane disintegration in GCs. However, when combined with the caspase-1-specific inhibitor VX-765 to suppress the classical pyroptotic pathway, the overall survival rate of KGN cells improved significantly, with a rescue effect markedly exceeding the additive effect expected from single-pathway inhibition alone (123, 124). This “double-blockade” experimental evidence reveals a synergistic mechanism involving parallel activation and mutual compensation between apoptosis and pyroptosis within the iatrogenic POI microenvironment, thereby providing direct mechanistic insight into the composite injury of ovarian GCs.

### 5.2 Pyroptosis and ferroptosis

Ferroptosis is characterised by the dysregulation of iron metabolism and uncontrolled lipid peroxidation as its core biochemical hallmarks. Within the POI microenvironment, ferroptosis and pyroptosis share common upstream molecular mechanisms that regulate ovarian GCs (118).

When the ovary is exposed to severe environmental toxins or age-related stress, glutathione peroxidase 4 (GPX4), the primary antioxidant defence system in GCs, frequently undergoes functional inactivation or degradation. Loss of GPX4 leads to massive cytosolic accumulation of lipid peroxides, such as L-OOH, and ROS (125). This intense oxidative stress response not only directly damages the plasma membrane but also triggers the initiation of the pyroptotic programme (Figure 2B). How does lipid peroxidation activate pyroptotic effector proteins? Recent studies have revealed that excessive intracellular ROS aberrantly activate members of the ZDHHC family of palmitoyl acyltransferases, particularly ZDHHC5 and ZDHHC9, which are localised to the plasma membrane and endosomal vesicles. Under oxidative stress conditions, these transmembrane enzymes precisely recognise the pyroptotic executor GSDMD and catalyse the covalent attachment of a 16-carbon lipophilic palmitate moiety to a specific and highly conserved cysteine residue on GSDMD (47). S-Palmitoylation fundamentally alters the biophysical properties of GSDMD. Following caspase-mediated cleavage, the lipid-modified N-terminal fragment of GSDMD exhibits markedly enhanced capacity for membrane translocation, accumulation, oligomerisation, and pore formation (47). These findings indicate that oxidative stress signals prevalent under ferroptotic conditions and pyroptosis do not occur independently; rather, they are tightly coupled through ZDHHC-mediated lipid modification.

In dying ovarian GCs, the interplay between GPX4 deficiency–induced lipid peroxidation and ZDHHC-driven GSDMD pore formation establishes a vicious cycle that ultimately accelerates complete follicular loss and ovarian fibrosis (126).

### 5.3 Pyroptosis and autophagy

Autophagy, particularly mitophagy, and the selective removal of damaged mitochondria, represents a critical protective mechanism by which ovarian GCs maintain cellular homeostasis, eliminate oxidative damage, and delay tissue ageing. Under physiological conditions, intact autophagic flux identifies, sequesters, and delivers dysfunctional mitochondria to lysosomes for degradation, thereby preventing mitochondrial constituents, such as oxidised mtDNA and excessive mtROS, from leaking into the cytoplasm and triggering innate immune responses. Studies have shown that, when autophagy-related proteins are impaired or autophagic flux is disrupted, free mtDNA escapes into the cytosol, where oxidized mtDNA functions as an endogenous danger-associated signal that promotes NLRP3 inflammasome activation, thereby triggering caspase-1 activation and subsequent GSDMD cleavage, ultimately culminating in inflammatory pyroptotic cell death (Figure 2C). This pathway—impaired autophagic flux → accumulation of damaged mitochondria and mtROS/oxidized mtDNA → NLRP3 activation → pyroptosis—has been extensively validated as a central mechanism in molecular immunology (68).

A representative example is CTX-induced POI. CTX induces loss of mitochondrial membrane potential, increased oxidative stress, and mitochondrial dysfunction in GCs. Concurrently, animal models commonly exhibit a characteristic pattern of “enhanced initiation but impaired degradation” of autophagy, as evidenced by increased LC3-II levels accompanied by the accumulation of p62/SQSTM1. This finding indicates that autophagosome formation remains intact or is enhanced, whereas autophagosome–lysosome fusion or lysosomal cargo degradation is impaired, resulting in defective autophagic flux (127, 128). Under these conditions, large amounts of damaged mitochondria and protein aggregates accumulate intracellularly, creating a permissive environment for the release of mtROS and oxidised mtDNA as well as aberrant activation of the NLRP3 inflammasome (129). Multiple studies using POI and CTX-induced models have demonstrated significant upregulation of NLRP3, caspase-1, GSDMD-N, IL-1β, and IL-18 following CTX treatment. Importantly, the restoration of autophagic flux or pharmacological inhibition of NLRP3 partially rescued ovarian histology and functional parameters, including follicle number, anti-Müllerian hormone (AMH) levels, and sex hormone profiles. Collectively, these *in vivo* and *in vitro* findings directly link autophagic arrest to pyroptosis-mediated ovarian injury (17, 129, 130).

The accumulation of p62/SQSTM1 is not only a classical biomarker of impaired autophagic flux but may also play an active regulatory role in amplifying inflammatory responses. As an autophagic receptor, p62 recognises ubiquitinated inflammasome complexes under conditions of intact autophagic flux and mediates their selective degradation through the autophagy–lysosome pathway, thereby limiting excessive activation of the NLRP3 inflammasome and subsequent IL-1β production (131). By contrast, when autophagic flux is disrupted, p62—being an autophagic substrate—is not efficiently cleared and consequently accumulates. This accumulation not only reflects defective autophagic function but may also promote inflammasome activation by enhancing pro-inflammatory signaling pathways (132). Indeed, elevated p62 levels have been associated with enhanced NLRP3–caspase-1 signaling, whereas p62 suppression attenuates inflammasome activation (127, 133). Moreover, inflammasome components such as ASC possess a remarkable capacity for self-oligomerisation, enabling the formation of higher-order supramolecular structures that provide the structural basis for inflammasome assembly and signal amplification (134, 135). Therefore, under conditions of autophagic impairment, sustained p62 accumulation serves not only as an indicator of autophagic dysfunction but also as a potential facilitator of persistent and propagating pyroptotic signaling through the modulation of inflammasome-associated signaling networks (136).

From a translational perspective, a deeper understanding of the crosstalk between pyroptosis and autophagy may provide valuable therapeutic insights. In POI models, such as CTX-induced ovarian injury, ovarian damage often manifests as a devastating “burnout” phenomenon, in which chemotherapeutic agents pathologically activate the mTOR signaling pathway, driving explosive and irreversible overactivation of dormant primordial follicles. Targeting the impaired autophagic flux, particularly defects in autophagosome maturation and lysosomal degradation, has demonstrated ovarian protective effects. For example, administration of rapamycin at 5 mg/kg every other day significantly suppresses CTX-induced mTOR activation, thereby limiting the excessive recruitment and depletion of primordial follicles at the source and effectively preserving the growing follicle pool (137). Another highly promising strategy is the direct interruption of the destructive inflammasome–pyroptosis cascade. In CTX-induced POI models, GCs typically exhibit profound impairment of energy metabolism, characterised by depletion of the essential metabolic cofactor NAD+. Recent experimental evidence has demonstrated that daily supplementation with NMN (500 mg/kg) effectively restores ovarian metabolic function. NMN administration markedly elevates intracellular NAD+ levels in gGCs, thereby activating the NAD+-dependent deacetylase SIRT2. Activated SIRT2 suppresses the NLRP3–caspase-1–GSDMD signaling axis by directly regulating the stability of the NLRP3 inflammasome (17). Consequently, NMN treatment significantly reduces ovarian levels of mature IL-1β, IL-18, and the pore-forming GSDMD-N fragment. This therapeutic strategy, which integrates metabolic restoration with anti-inflammatory intervention, translates directly into increased follicle counts and an improved ovarian index. Collectively, these findings suggest that replenishing the intracellular “energy currency” (NAD+) and restoring the “waste clearance” capacity of the autophagic system may exert synergistic and complementary effects on ovarian functional reconstruction (138).

## 6 Potential therapeutic strategies targeting pyroptosis in POI

With a deeper understanding of the molecular mechanisms underlying POI, it has become increasingly evident that strategies that rely solely on hormone replacement or anti-apoptotic interventions are often insufficient to reverse the progressive decline in ovarian reserve. Accumulating evidence in recent years has demonstrated that inflammasome-mediated pyroptosis plays a pivotal role in GC injury and follicular cell loss. Accordingly, therapeutic interventions targeting key nodes of the pyroptotic pathway, particularly mitochondrial oxidative stress, NLRP3 inflammasome activation, and GSDMD activation, are emerging as promising avenues for POI treatment. To facilitate comparison of these emerging therapeutic approaches and clarify their translational potential, we summarize representative pyroptosis-targeting interventions, their molecular targets, mechanisms, and current evidence levels in Table 1.

**Table 1:** Summary of therapeutic strategies targeting pyroptosis-associated pathways in premature ovarian insufficiency.

Agent/Strategy	Molecular targe	Mechanism	Evidence level	Key findings in POI/inflammation models	Translational status	References
MitoQ	Mitochondrial ROS	Scavenges mtROS and restores mitochondrial homeostasis	*In vivo*	Reduces oxidative stress, suppresses inflammasome activation, protects ovarian function	Preclinical	(140, 141)
MCC950	NLRP3 inflammasome	Selective inhibition of NLRP3 activation	*In vitro*/*in vivo*	Suppresses NLRP3-caspase-1-GSDMD axis	Clinical development limited	(115)
VX-765	Caspase-1	Inhibits caspase-1 enzymatic activity	*In vitro*/*in vivo*	Reduces IL-1β maturation and pyroptosis	Clinical trials in inflammatory diseases	(146)
Disulfiram	GSDMD/NLRP3	Inhibits pyroptosis-related pore formation and inflammasome activation	*In vitro*/*in vivo*	Reduces pyroptosis and inflammatory injury	Approved drug, repurposing potential	(160)
2-BP	ZDHHC-mediated palmitoylation	Blocks protein S-palmitoylation	*In vitro*	Reduces GSDMD membrane localization and pyroptosis	Experimental	(47)
NMN	NAD+/SIRT pathway	Restores mitochondrial metabolism and inhibits NLRP3 signaling	*In vivo*	Improves ovarian reserve markers in CTX-POI models	Nutraceutical/clinical exploration	(17)
Rapamycin	mTOR/autophagy pathway	Restores autophagic regulation and prevents follicle burnout	*In vivo*	Preserves primordial follicle pool in CTX models	Clinical use in other diseases	(137)
PROTACs	Targeted protein degradation	Induces selective degradation of pathogenic proteins	Conceptual/preclinical	Potential regulation of inflammasome components	Early-stage development	(143, 144)
MSC-derived exosomes	Multiple pathways	Delivers anti-inflammatory/anti-oxidative cargo	*In vivo*	Improves ovarian injury models and reduces inflammatory cell death	Translational research	(88, 153)

### 6.1 Targeting mitochondrial homeostasis and excessive ROS accumulation

Mitochondrial dysfunction and excessive ROS production are considered critical upstream events that trigger ovarian inflammation and activate pyroptotic signaling. As highly active endocrine cells, ovarian GCs depend heavily on mitochondrial oxidative phosphorylation to sustain ATP production and steroid hormone biosynthesis. Consequently, following exposure to chemotherapeutic agents, environmental toxicants, or physiological processes associated with ageing, GCs frequently undergo an irreversible decline in mitochondrial membrane potential. Under these conditions, excessive mtROS accumulation reflects mitochondrial dysfunction and can further exacerbate mitochondrial damage, promoting the release of ox-mtDNA and other mitochondrial danger-associated molecules. These endogenous danger signals subsequently facilitate pattern-recognition receptor activation and NLRP3 inflammasome assembly (68, 70, 139). mtROS, alongside mtROS-induced oxidative stress and intermediate signals (such as ox-mtDNA leakage and TXNIP dissociation), promote molecular events that facilitate NLRP3 activation, leading to its higher-order oligomerization with the adaptor protein ASC, which subsequently induces autocleavage and activation of caspase-1. Activated caspase-1 subsequently cleaves GSDMD, ultimately leading to the pyroptotic death of GCs (70). Based on this mechanistic framework, therapeutic strategies aimed at mitigating mitochondrial oxidative stress have attracted increasing attention. The mitochondria-targeted antioxidant MitoQ selectively accumulates within the mitochondria in a membrane potential-dependent manner, where it reduces mtROS levels by scavenging superoxide anions and hydrogen peroxide. Animal studies have demonstrated that MitoQ effectively attenuates oxidative stress-induced tissue inflammation and suppresses the activation of inflammasome-associated signaling pathways (140). In ovarian injury models, antioxidants, including mitochondria-targeted agents such as MitoQ and classical precursor antioxidants such as N-acetylcysteine, have been shown to reduce ROS levels, decrease GC death, and partially restore follicle number and hormone secretory function (141). Collectively, these findings suggest that restoration of mitochondrial homeostasis and suppression of excessive ROS generation may represent promising strategies for interrupting sustained inflammasome activation at an early stage of the pyroptotic signaling cascade.

### 6.2 Therapeutic strategies targeting PTMs of pyroptosis

#### 6.2.1 Targeting palmitoylation

S-palmitoylation plays a dual regulatory role in the pyroptotic pathway by promoting the interaction between upstream NLRP3 and NEK7, while also enhancing the membrane localisation and oligomerisation of the downstream effector GSDMD-N. The palmitoylation inhibitor 2-bromopalmitate (2-BP) has been widely used in pyroptosis-related studies to suppress protein palmitoylation. Previous studies have demonstrated that 2-BP effectively inhibits inflammasome-induced membrane translocation of GSDMD and subsequent pyroptosis, thereby reducing the release of IL-1β and IL-18 (47). In ovarian injury models, inhibition of NLRP3 inflammasome assembly together with the suppression of GSDMD membrane localisation or pore formation may reduce the inflammatory death of GCs and delay follicular atresia. Therefore, the development of therapeutic agents targeting the ZDHHC–GSDMD axis possesses considerable translational potential.

Notably, critical lipid modification sites in both GSDMD and NLRP3, such as Cys191 in human GSDMD, are reversible. In addition, cysteine residues are capable of undergoing multiple alternative PTMs. It may therefore be hypothesised that modulation of competitive PTMs at these intracellular cysteine residues may induce alternative modifications at the same critical sites, thereby generating steric hindrance and competitive antagonism against pathogenic S-palmitoylation. This site competition–based “modification substitution” mechanism offers a highly promising conceptual framework for the development of structurally specific inhibitors that selectively block pathogenic palmitoylation.

#### 6.2.2 Targeting the ubiquitination system

The ubiquitin–proteasome system (UPS), a central regulator of intracellular proteostasis, plays an indispensable role in controlling the inflammasome activation threshold. Under physiological conditions, multiple endogenous E3 ubiquitin ligases effectively restrain excessive inflammasome activation by attaching specific “degradation tags” to core components of the pyroptotic machinery.

With the rapid development of proteolysis-targeting chimaeras (PROTACs), researchers have begun to exploit the endogenous UPS to achieve selective degradation of pathogenic proteins, thereby shifting therapeutic strategies from conventional passive signal inhibition towards targeted protein elimination (142). Unlike reversible small-molecule inhibitors that act primarily through occupancy of catalytic or binding sites, PROTAC molecules induce degradation of target proteins, thereby producing more sustained pharmacological effects. PROTACs are typically composed of two functional modules: one binds to the target protein, whereas the other recruits an intracellular E3 ubiquitin ligase, such as cereblon or von Hippel–Lindau, thereby promoting the formation of a stable ternary complex in spatial proximity. A recently reported lead PROTAC degrader targeting NLRP3, NP-16, exemplifies this strategy. By simultaneously binding NLRP3 and recruiting an E3 ubiquitin ligase, this class of molecules induces polyubiquitination of the target protein, leading to its recognition and degradation by the proteasome, thereby effectively suppressing inflammasome signaling activity (143). In principle, this proteostasis-directed degradation strategy could reduce the functional abundance of key pyroptotic effector proteins such as GSDMD at the source, thereby suppressing execution of the pyroptotic pathway. Although the development of PROTAC-based therapeutics targeting reproductive system–related proteins remains at an exploratory stage, this approach has already demonstrated more durable pathway suppression than conventional inhibitors in studies on autoimmune diseases and tumour microenvironment regulation. These findings suggest considerable translational potential for future interventions targeting chronic inflammatory ovarian injury associated with POI (144).

Overall, current evidence suggests clear functional divergence in ubiquitination-mediated regulation of GSDMD. One class of E3 ubiquitin ligases enhances GSDMD-mediated membrane pore formation by promoting ubiquitination, whereas another limits pyroptotic signaling by inducing its degradation. However, compared with the ubiquitin-dependent regulation of upstream inflammasome components, studies focusing on E3 ligases that directly regulate GSDMD remain relatively limited, particularly in ovarian GCs and POI-related models, in which the underlying mechanisms remain largely unexplored. Future systematic identification of GSDMD-specific E3 ubiquitin ligases, together with clarification of their cell type–specific functional heterogeneity, will provide an important theoretical foundation for the development of precise therapeutic strategies targeting pyroptosis.

#### 6.2.3 Targeting phosphorylation cascades

The phosphorylation status of core pyroptotic proteins directly determines their interaction affinity and activation threshold, thereby providing precise pharmacological targets. Given the bidirectional regulatory nature of phosphorylation, therapeutic strategies can be broadly categorised into inhibition of pro-inflammatory kinases and activation of protective kinases.

During the upstream sensing stage, PKD promotes inflammasome assembly through phosphorylation of NLRP3 and NEK7. Studies have demonstrated that highly selective small-molecule inhibitors of PKD, such as CRT0066101, or JNK1 inhibitors, such as SP600125, can interrupt the signaling cascade at an early amplification stage of pyroptosis, thereby preventing pathogenic conformational changes in NLRP3 (60, 61).

During the downstream execution stage, metabolic sensing kinases such as AMPK can exert a protective “molecular lock” on pyroptotic effector proteins. AMPK directly targets and phosphorylates GSDMD at Ser46, severely impairing its oligomerisation and pore-forming capacity at the structural level (59). Consequently, when GCs are exposed to chemotherapeutic injury or metabolic stress, the use of AMPK-specific activators—such as metformin or AICAR—may not only restore cellular energy homeostasis but also effectively block the terminal execution step of pyroptosis. Similarly, activation of PKA via adenylate cyclase agonists, such as forskolin, which elevate intracellular cyclic AMP levels, can induce inhibitory phosphorylation of NLRP3 at Ser295. This represents a potentially powerful strategy for suppressing inflammatory storm–like responses in ovarian cells (63).

Overall, intervention strategies targeting three major PTMs—palmitoylation, ubiquitination, and phosphorylation—enable multilayered regulation along the continuous axis of “inflammasome activation–GSDMD execution”. By focusing on modulation of protein function rather than merely suppressing protein expression, these approaches may help avoid adverse effects associated with broad immunosuppression while also providing an important theoretical foundation for the development of highly selective and reversible anti-pyroptotic therapeutic strategies.

### 6.3 Classical inhibitors targeting the inflammasome pathway

Within the complex cascade regulatory network of pyroptosis, direct intervention at the level of inflammasome assembly and its downstream effector molecules is currently regarded as one of the most well-established therapeutic strategies in translational medicine, as supported by robust pharmacological evidence. By pharmacologically blocking key hubs within this signaling pathway, inflammatory amplification can be suppressed either at its origin or during the execution phase, thereby mitigating tissue injury.

As a core PRR in the inflammasome pathway, NLRP3 oligomerisation is a critical step in inflammasome assembly and the initiation of pyroptosis. The small-molecule diarylsulfonylurea MCC950 is currently one of the most extensively studied and highly selective NLRP3 inhibitors. Unlike conventional broad-spectrum anti-inflammatory agents, MCC950 selectively binds to the NACHT domain of the NLRP3 protein and non-competitively inhibits its ATP hydrolysis activity, thereby stabilising NLRP3 in an inactive closed conformation and preventing its oligomerisation. This mechanism structurally disrupts the interaction between NLRP3 and the adaptor protein ASC, ultimately blocking inflammasome complex assembly and downstream signal transduction (145).

In studies related to POI, MCC950 has demonstrated pronounced ovarian protective effects. Multiple animal studies have shown that, in CTX-induced POI models, pharmacological intervention with MCC950 significantly attenuates local ovarian inflammatory responses, markedly reduces IL-1β and IL-18 levels, and effectively suppresses GC pyroptosis. Histological analyses further indicated that MCC950 treatment significantly reduced follicular atresia, preserved a greater number of primordial and growing follicles, and partially restored serum hormone levels and reproductive capacity (76).

In addition to direct inhibition of upstream inflammasome sensors, targeting key effector enzymes involved in the execution of pyroptosis represents another important therapeutic strategy. Caspase-1 is a central executioner protease within the inflammasome signaling pathway. The prodrug VX-765 (belnacasan) is a well-characterised caspase-1 inhibitor that is rapidly converted intracellularly into its active metabolite, VRT-043198. This active compound covalently binds to the catalytic cysteine residue within the active site of caspase-1, thereby inhibiting its proteolytic activity and blocking the maturation of proinflammatory cytokines (146, 147).

In multiple inflammation-associated ovarian dysfunction models, including POI and PCOS accompanied by GC pyroptosis, both *in vivo* and *in vitro* studies have confirmed that VX-765 significantly inhibits the conversion of pro–IL-1β to mature IL-1β. In addition, owing to the suppression of caspase-1 activity, GSDMD cannot be cleaved into its N-terminal fragment and therefore remains in its inactive full-length form within the cell, effectively blocking the execution phase of pyroptosis. Through pharmacological inhibition of key intermediate nodes in the inflammatory signaling pathway, VX-765 improves the local ovarian inflammatory microenvironment and reduces follicular atresia, thereby providing experimental evidence supporting the development of non-hormonal ovarian protective strategies (148–150).

### 6.4 Mesenchymal stem cells (MSCs) and their exosome-based therapy

In the fields of tissue repair and regenerative medicine, MSCs and their cell-free derivatives have emerged as important therapeutic candidates for ovarian dysfunction. An increasing body of evidence indicates that the therapeutic effects of MSCs do not primarily depend on their homing to injured tissues or their differentiation capacity but are instead mainly mediated through potent paracrine mechanisms. MSCs continuously secrete a variety of bioactive molecules into the local microenvironment, including growth factors, antioxidant enzymes, and regulatory microRNAs (miRNAs), thereby modulating local immune responses, alleviating oxidative stress, and promoting tissue repair and regeneration (151). In multiple animal studies, intravenous or local administration of MSCs has been shown to significantly improve the histological structure of atrophic ovaries, increase the primordial follicle reserve, and restore secretion of oestrogen and AMH, thereby partially reversing ovarian functional decline (152).

In recent years, MSC-derived exosomes (MSC-Exos) have emerged as key mediators of these therapeutic effects owing to their favourable biocompatibility and low immunogenicity. Exosomes are lipid bilayer nanovesicles of approximately 30–150 nm in diameter that can carry proteins, lipids, and various regulatory RNA molecules and participate in a wide range of pathophysiological processes through intercellular communication. Accumulating evidence suggests that MSC-Exos can deliver their cargoes of miRNAs and antioxidant-related proteins to damaged ovarian GCs, thereby modulating cellular stress responses and inflammatory signaling pathways. On the one hand, these bioactive molecules reduce mtROS levels and restore mitochondrial membrane potential homeostasis. On the other hand, specific miRNAs can suppress the expression of NLRP3 inflammasome–related molecules at the post-transcriptional level, thereby attenuating inflammatory responses and pyroptotic signaling 3. In CTX-induced POI models, as well as autoimmune ovarian injury models, administration of hUCMSC-Exos has been shown to significantly reduce the expression of NLRP3, activated caspase-1, and mature IL-1β in ovarian tissues while simultaneously inhibiting GSDMD-mediated pyroptosis (88, 153). These molecular alterations ultimately translate into histological and functional improvements, including restoration of follicular development and reduced GC death. Overall, MSC-Exos represent a promising cell-free therapeutic strategy for restoring ovarian function in patients with POI through regulation of mitochondrial homeostasis and suppression of inflammasome-mediated pyroptosis.

### 6.5 Challenges and prospects in the clinical translation of pyroptosis-targeted strategies for POI

Although intervention strategies targeting the pyroptosis pathway have shown promising therapeutic effects in cellular and animal models, their translation into clinical practice remains hindered by several substantial hurdles.

#### 6.5.1 Systemic immunosuppression and safety risks

Core execution and regulatory molecules of pyroptosis—such as NLRP3, caspase-1, and GSDMD—are integral components of the host's innate immune defense system. Consequently, systemic or prolonged inhibition of these targets may compromise host clearance of intracellular pathogens, thereby heightening susceptibility to severe infections (154). Furthermore, as pyroptosis serves as a crucial effector mechanism through which CD8+ T cells and NK cells exert anti-tumor immune surveillance, long-term blockade of pyroptotic pathways carries a potential risk of facilitating tumor immune evasion (41, 155). Illustratively, while the canonical NLRP3 inhibitor MCC950 exhibited profound therapeutic efficacy in animal models, its early-phase clinical development was halted due to unexpected hepatotoxicity (115). Thus, achieving robust suppression of localized pathogenic pyroptosis in the ovary without compromising systemic immune homeostasis represents a paramount safety challenge for clinical evaluation.

#### 6.5.2 Targeted delivery and ovarian biodistribution

As a deep pelvic organ, the ovary is safeguarded by the blood–follicle barrier (BFB)—composed of vascular endothelium and surrounding granulosa cell layers (156). Consequently, conventional systemic administration yields sub-therapeutic intra-ovarian drug bioavailability, while simultaneously escalating off-target exposure and systemic toxicity (156). To overcome these limitations, advanced delivery platforms—such as smart nanocarriers functionalized to target granulosa cell surface receptors (157) or mesenchymal stem cell-derived exosomes (MSC-Exos) leveraging intrinsic lesion-homing capacities (153)—are urgently required to achieve precise accumulation and controlled release of anti-pyroptotic agents specifically within the injured ovarian microenvironment.

#### 6.5.3 Patient stratification and therapeutic timing

POI is a highly heterogeneous disorder with diverse etiologies, including iatrogenic injury, autoimmune destruction, genetic defects, and environmental exposures (158). Consequently, pyroptotic magnitude and its key molecular drivers vary substantially across clinical phenotypes (116). Because pyroptosis primarily executes acute, early-stage follicular damage and atresia, anti-pyroptotic monotherapy cannot induce *de novo* folliculogenesis in end-stage POI where the primordial pool is virtually exhausted (159). Therefore, integrating pyroptotic cytokines (notably IL-1β and IL-18) with standard ovarian reserve parameters is essential to establish an actionable therapeutic window—specifically identifying patients in a hyper-pyroptotic state who retain salvageable primordial follicles to maximize clinical efficacy (110). Importantly, despite extensive preclinical evidence, no pyroptosis-targeting agent has yet been approved specifically for POI treatment, and most candidate interventions remain at the experimental or early translational stage. As mentioned earlier, the presence of liver toxicity in MCC950 has restricted its clinical development, highlighting the importance of conducting a comprehensive toxicity assessment before its use in reproductive applications. In contrast, disulfiram, an FDA-approved drug originally used for alcohol dependence, has recently attracted attention because of its ability to inhibit NLRP3 inflammasome activation and pyroptosis through regulation of protein palmitoylation, although its efficacy and safety in reproductive disorders remain untested (160). Similarly, IL-1β pathway inhibitors, including anakinra and canakinumab, have entered clinical practice for inflammatory diseases, providing indirect evidence that targeting downstream inflammatory mediators may be clinically feasible; however, whether these approaches can preserve ovarian reserve or restore fertility requires dedicated clinical investigation (161).

Collectively, future clinical translation of pyroptosis-based therapies for POI will require multidisciplinary efforts integrating disease-specific biomarkers, ovarian-targeted delivery systems, rigorous reproductive safety assessment, and well-designed clinical trials. Establishing a precise therapeutic window that suppresses pathological pyroptosis while preserving physiological immune functions will be essential for converting promising experimental findings into clinically applicable interventions.

## 7 Conclusion

### 7.1 Critical role of pyroptosis in the pathogenesis of POI

In recent years, with a deeper understanding of programmed cell death networks, pyroptosis has extended beyond its traditional role in immunological research and is now recognised as a critical molecular hub linking ovarian microenvironmental injury, chronic inflammatory responses, and irreversible follicular depletion. Traditional perspectives in reproductive endocrinology have suggested that the principal pathological basis of POI is the excessive apoptosis of ovarian parenchymal cells. However, emerging evidence suggests that inflammasome-mediated pyroptosis is a key driver of disrupted ovarian microenvironmental homeostasis (118). In this process, excessive activation of the NLRP3 inflammasome triggers caspase-1 activation, leading to GSDMD cleavage and the release of its pore-forming N-terminal fragment. The N-terminal fragment of GSDMD subsequently forms transmembrane pores in the plasma membrane, disrupts membrane integrity, and mediates the massive release of pro-inflammatory cytokines such as IL-1β and IL-18, thereby further amplifying local inflammatory responses (27, 67).

Within the complex architecture of ovarian tissue, GCs serve as essential supporting cells for oocyte growth and follicular development and are regarded as key components of the microenvironment governing oocyte metabolism and endocrine regulation. When GCs undergo inflammatory pyroptosis, not only is their nutritional and signaling support for the oocyte disrupted, but paracrine effects triggered by the release of pro-inflammatory mediators may further accelerate follicular atresia and lead to a marked decline in the ovarian reserve (118, 129, 162). In CTX-induced POI mouse models, not only was a significant reduction in primordial follicle number observed, but expression levels of NLRP3, activated caspase-1, and GSDMD-N were also markedly elevated, accompanied by significantly increased IL-1β levels in both ovarian tissue and serum (162, 163). These findings suggest that sustained aberrant activation of pyroptosis-related signaling pathways may play a central role in the initiation and progression of POI rather than merely representing a secondary consequence of tissue injury.

### 7.2 Current limitations of existing research

Although the number of studies investigating the role of pyroptosis in ovarian injury and POI has increased substantially in recent years, translation of these findings from basic research into clinical application continues to face numerous challenges and limitations. As summarized in Table 1, most pyroptosis-targeting strategies remain at the preclinical stage, with only a limited number of agents having entered clinical evaluation for inflammatory disorders unrelated to POI.

A major limitation of current research lies in the experimental models employed. At present, most studies investigating ovarian pyroptosis primarily rely on single GC models cultured in conventional two-dimensional *in vitro* systems or rodent models of POI induced by chemotherapeutic agents such as cyclophosphamide and cisplatin. To some extent, these models can recapitulate the characteristic pathological features of POI, including depletion of the follicular reserve and enhanced local ovarian inflammatory responses. However, substantial interspecies differences exist between mice and humans in terms of reproductive ageing, ovarian tissue architecture, and neuroendocrine–immune regulatory networks. Therefore, caution is required when extrapolating findings derived from animal models to human diseases (164, 165). In addition, owing to ethical constraints and limited clinical accessibility, obtaining human ovarian tissue samples remains difficult, particularly from patients with early-stage POI. As a result, pyroptosis-related multi-omics data and *in situ* molecular evidence in human ovarian tissue remain insufficient, limiting further mechanistic investigations.

Current studies on the mechanisms underlying pyroptosis in POI exhibit certain conceptual limitations. Existing research has focused predominantly on the canonical NLRP3–caspase-1–GSDMD inflammasome signaling axis. However, regulatory networks governing programmed cell death are complex. For example, the non-canonical inflammasome pathway plays a critical role in innate immune responses and inflammatory stress. This pathway recognises intracellular danger signals, such as LPS, and directly activates caspase-4/5 in humans or caspase-11 in mice, thereby inducing GSDMD cleavage, promoting membrane pore formation, and ultimately triggering inflammatory cell death (27, 28).

GSDME, a member of the GSDM family, also plays an important role in regulating the switch between distinct modes of cell death. Previous studies have shown that, under chemotherapeutic or other stress-related stimuli, the apoptotic executioner caspase-3 specifically cleaves GSDME, thereby converting apoptosis, which is relatively immunologically silent, into a pyroptotic process characterised by pronounced inflammatory responses (40). This apoptosis-to-pyroptosis switch has been well established in tumour biology and chemotherapy-associated toxicity studies. Given that chemotherapeutic agents are the major iatrogenic triggers of POI, GSDME-mediated switching of cell death may play a role in ovarian follicular injury and follicular atresia. However, systematic investigations into the specific roles of non-canonical pyroptosis pathways, such as the caspase-11 axis and GSDME-mediated apoptosis–pyroptosis crosstalk, in the initiation and progression of POI remain lacking. Their precise contribution to ovarian pathology warrants further investigation.

### 7.3 Future research directions and translational perspectives

Future investigations into the mechanisms of pyroptosis in POI should adopt deeper, multidisciplinary, and multilevel approaches to facilitate effective translation of basic research findings into clinical diagnostic and therapeutic strategies.

An important future direction is the development of a non-invasive early warning system based on liquid biopsy. Because the ovarian tissue is located deep within the pelvic cavity, conventional tissue biopsy is highly invasive and difficult to perform repeatedly, underscoring the importance of identifying reliable biofluid biomarkers. In recent years, circulating exosomes have attracted considerable attention as promising biomarkers because they can stably carry molecular information derived from their cells of origin, including proteins, lipids, and miRNAs. Consequently, they are considered important mediators reflecting alterations in the tissue microenvironment. Studies have shown that exosomal miRNA expression profiles can, to some extent, reflect the functional status of ovarian GCs and the degree of inflammatory injury (108, 109). Therefore, future large-scale clinical cohort studies are warranted to systematically identify exosome-derived molecular biomarkers closely associated with pyroptosis-related signaling pathways, such as the NLRP3 inflammasome axis, including specific miRNA subsets or GSDMD-related protein fragments. Such efforts may facilitate the establishment of a biomarker system for POI with greater early predictive value than conventional indicators of ovarian reserve, such as AMH.

With the continuous development of small-molecule inhibitors targeting pyroptosis-related signaling pathways, evaluating their long-term safety in the reproductive system has become a critical issue in future translational research. For example, the selective NLRP3 inflammasome inhibitor MCC950 has demonstrated significant anti-inflammatory effects in multiple animal models of inflammatory disease (145). However, the ovary itself is characterised by a certain degree of localised inflammatory activity. For instance, ovulation is widely regarded as an inflammation-like physiological process. In this context, whether long-term or systemic inhibition of the NLRP3 or caspase-1 signaling pathways adversely affects key reproductive processes, including follicular development, ovulation, and corpus luteum formation, remains to be systematically evaluated through reproductive toxicology studies and long-term animal experiments (76).

Regenerative medicine strategies have also opened new avenues for the treatment of POI. Recently, MSCs and MSC-Exos have demonstrated promising immunomodulatory and tissue reparative capacities in various tissue injury and inflammatory disease models (107). In disease-specific POI models, highly purified MSC-Exos can effectively penetrate the damaged ovarian parenchyma. Through targeted delivery of antioxidant proteins and regulatory miRNAs enriched within these vesicles, MSC-Exos can markedly suppress local inflammatory cascades. Accumulating evidence indicates that this exosome-based targeted intervention not only improves the pathological microenvironment of the ovarian stroma and effectively attenuates inflammatory death of GCs but also promotes the reactivation of follicular development at multiple stages and restores the sex hormone secretor*y* axis, thereby substantially reversing the progressive decline in ovarian function (152). In the future, combining MSC-Exos–based therapy with novel biomaterial delivery systems, such as biodegradable hydrogels or nanocarrier platforms, to achieve precise local delivery and sustained release within the ovary may provide a more effective therapeutic strategy for the clinical management of POI.

### 7.4 Future perspectives

In summary, identification of pyroptosis has provided a novel perspective for elucidating the pathological inflammatory mechanisms underlying POI, thereby extending beyond the traditional single-pathway model centred primarily on apoptosis. With the rapid development of single-cell RNA sequencing, spatial omics, and integrative multi-omics analytical approaches, researchers are now able to characterise the molecular features and dynamic evolution of distinct cellular populations within ovarian tissue at a substantially higher resolution (166). In the future, deep integration of multi-omics technologies with computational biology approaches is expected to further elucidate the spatiotemporal regulatory landscape of ovarian pyroptosis, thereby providing a more precise theoretical foundation for the early diagnosis and targeted treatment of POI and ultimately advancing reproductive medicine toward more precise and individualised therapeutic strategies.
